# The speed and temporal frequency of visual apparent motion modulate auditory duration perception

**DOI:** 10.1038/s41598-023-38183-w

**Published:** 2023-07-12

**Authors:** Xiang He, Zijun Ke, Zehua Wu, Lihan Chen, Zhenzhu Yue

**Affiliations:** 1 Department of Psychology, Sun Yat-Sen University, Guangzhou, 510006 China; 2 School of Psychological and Cognitive Sciences and Beijing Key Laboratory of Behavior and Mental Health, Peking University, Beijing, 100871 China

**Keywords:** Psychology, Perception

## Abstract

In the present study, we investigated how the perception of auditory duration could be modulated by a task-irrelevant, concurrent visual apparent motion, induced by visual bars alternating between left and right sides. Moreover, we examined the influence of the speed and temporal frequency of visual apparent motion on the perception of auditory duration. In each trial, the standard visual stimuli (two vertical bars) were presented sequentially, except that visual apparent motion was included in the fourth stimulus. A tone was presented simultaneously with each visual stimulus, while the fourth tone was presented with varied duration. Participants judged whether the fourth tone lasted longer than the other four tones. In Experiment 1, the speed of visual apparent motion (Fast vs. Slow) was manipulated by changing the interval between two bars. The mean point of subjective equality (PSE) in the Slow apparent motion condition was larger than that in the Static condition. Moreover, participants tended to overestimate the duration only in the Static condition, i.e., time dilation effect, which disappeared under apparent motion conditions. In Experiment 2, in addition to speed, we controlled the temporal frequency of apparent motion by manipulating the number of bars, generating four conditions of visual apparent motion (Physical-fast, Perceived-fast, Perceived-slow, vs. Static). The mean PSE was significantly smaller in the Physical-fast condition than in the Static and Perceived-slow conditions. Moreover, we found a time compression effect in both the Perceived-slow and Static conditions but not in the Perceived-fast and Physical-fast conditions. These results suggest that the auditory duration could be modulated by the concurrent, contextual visual apparent motion, and both the speed and temporal frequency of the task-irrelevant visual apparent motion contribute to the bias in perceiving the auditory duration.

## Introduction

Time perception is the ability to perceive timing, which plays a vital role in our daily life. Researchers have proposed the scalar expectancy theory (SET)^1,2^ to explain the processing of temporal information, which holds the view that time perception is based on the output of an internal clock. In particular, the pacemaker generates and delivers pulses to the accumulator. The number of pulses is used as a reference for temporal judgement. The more stimuli there are, the more accumulated pulses. Therefore, people will perceive the duration as longer when more pulses are accumulated and vice versa. In short, SET suggests that time perception is the output of a specialized pacemaker-accumulator processing system, which means that more visual bars would generate more pulses to the accumulator and thus lead to longer time perception.

A few empirical studies have shown that subjective time perception can be distorted by task-irrelevant sensory properties, including stimulus size^3,4^, spatial frequency^5^, and individual emotional states^6^. For example, the duration of larger and brighter stimuli is generally perceived as longer^4,5^. In addition, time perception could also be modulated by the continuity of stimuli. For example, previous studies found that people tended to overestimate the duration of discontinuous stimuli (e.g., visual flickers or auditory flutters) relative to continuous stimuli^7^. In addition to the sensory properties mentioned above, motion has been demonstrated to affect the perceived duration of visual stimuli. Generally, the duration of moving objects is perceived to be longer than that of stationary stimuli^8–12^. For example, Gorea and Kim found that participants overestimated the time duration of Gabor patch movement^10^.

Some researchers have found that time perception in one modality could be biased by nontemporal factors from another modality^13–20^. Therefore, there remains a debate regarding whether the bias of time perception is modality-specific or modality-general. Barne et al. suggested that there existed a common neural representation for processing time intervals, i.e., a supra-modal timing mechanism. In their study, participants were required to reproduce intervals from 750 to 1500 ms marked by auditory or visual stimuli. By using multivariate pattern analysis (MVPA) in scalp electroencephalogram (EEG), they found that a similar pattern of EEG activities was observed for visual and auditory time intervals^16^. In contrast, some studies have suggested two distinct, modality-specific mechanisms for auditory and visual time processing. For example, by adopting a cross-modal oddball paradigm, Chen et al. required participants to complete two oddball tasks (attended to visual or auditory modality)^19^. The standard stimulus was a 200 ms red circle or 1000 Hz sinusoidal tone, while the deviant stimulus was the same type of stimulus but lasted 120 ms. They found that the visual mismatch negativity (MMN) was significantly larger in the attended condition than in the unattended condition over the frontal-central sites, while the auditory MMN was not modulated by attention. Similarly, Bratzke and Ulrich adopted a temporal reproduction task in which standard stimuli (white noises or blue squares, 800 vs. 2400 ms) were presented, and participants were asked to reproduce the duration within the same modality or across different modalities. They found that performance was better in the congruent condition (e.g., two durations from the same modality) than in the incongruent condition (e.g., two durations from different modalities)^20^.

Among all the possible modulating factors of the temporal bias, the speed and the temporal frequency of the distractors are critical modulating factors. However, the exact contribution of the two factors remains unclear. On the one hand, previous studies have provided evidence that speed is the key factor for temporal perception. For example, Kaneko and Murakami manipulated the temporal frequency (0, 1, 2, 4, 8, and 16 Hz) and spatial frequency (0.5, 1, 2, and 4 c/deg) and defined the speed as the temporal frequency divided by the spatial frequency. The results indicated that the perceived duration of the Gabor patch was mainly affected by the speed rather than the temporal frequency of the motion stimuli^9^. On the other hand, other studies have supported temporal frequency is important for temporal perception. For example, Kanai et al. used expanding gratings as stimuli and found that people perceived a longer time duration in high frequency conditions (4.0 Hz) than in the low-frequency condition (0.5 Hz)^12^. Previous studies support that the processing of visual apparent motion activates similar brain regions as real motion^21–23^; however, it remains unclear whether apparent motion can distort duration perception.

The primary objective of this study was to examine if perception of target duration in one sensory modality could be influenced by task-irrelevant apparent motion in a different modality. We explored whether visual apparent motion could impact duration perception in another modality, such as auditory duration. For visual apparent motion, we used stimuli similar to those in Freeman and Driver's study^24^. In the first experiment, we investigated the potential modulation of auditory time perception by concurrent task-irrelevant visual apparent motion. By presenting two alternating bars with varying empty intervals, we manipulated speed (Fast vs. Slow). Additionally, we used two static bars separated by a distance as control stimuli. We hypothesized that time duration would be perceived as longer in apparent motion conditions compared to the static condition, with a more pronounced distortion in the fast apparent motion condition. In the second experiment, we altered the temporal frequency of apparent motion by adjusting the number of bars, in addition to speed. We hypothesized that participants would perceive time duration as longer in the high-frequency condition compared to the low-frequency condition^12^.

## Experiment 1

In this experiment, we aimed to investigate how simultaneous visual apparent motion modulated the perception of auditory duration. Participants were required to compare auditory durations, while visual static stimuli or visual apparent motion were presented simultaneously.

### Methods

#### Participants

Seventeen college students (9 males, mean age 22 years, range 20–24 years, SD 1.65 years) took part in the experiment. All participants reported normal hearing and normal or corrected-to-normal vision acuity. Participants were compensated after the experiment. All participants signed informed consent before the experiment. The study was conducted in accordance with the guidelines in the Declaration of Helsinki (2000) and approved by the Ethics Committee of the Department of Psychology, Sun Yat-sen University.

#### Stimuli and apparatus

**Figure 1:**
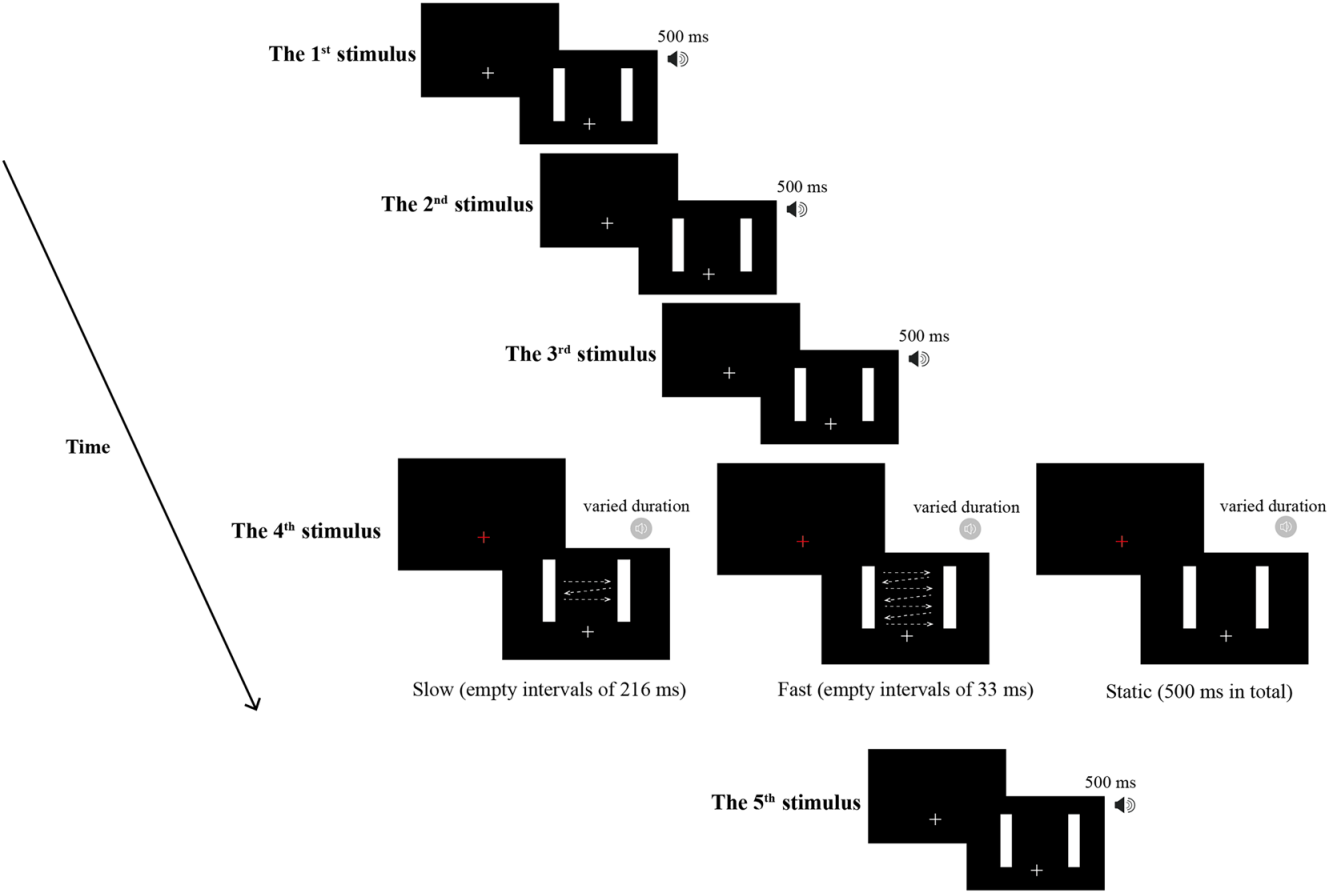
Trial sequence in Experiment 1. In each trial, the standard stimuli (two vertical white bars) were presented sequentially, except that visual apparent motion are included in the fourth stimulus. Five successive tones were presented simultaneously with each visual stimulus, except the fourth tone with varied duration. Participants judged whether the fourth tone lasted longer than the other four tones. For the apparent motion stimulus, left or right bar appeared in alternation for 30 ms with empty intervals of 216 ms (Slow apparent motion) or 33 ms (Fast apparent motion).

Visual stimuli were presented on a DELL monitor (27 inch, 1600 × 900 pixels resolution, refresh rate: 60 Hz) and controlled by E-prime2.0 software (https://pstnet.com/products/e-prime/). Two vertical bars (width of 1° visual angle) were presented, separated by a distance of 17° visual angle. The auditory stimuli were sampled at 44.1 kHz and quantized to 16 bits, which were generated by Adobe Audition 3.0 software (https://www.adobe.com/cn/products/audition.html) and presented via a headphone (Sony MDR-xb4500). In each trial, a sequence of visual bars and auditory tones were presented. For the 1st, 2nd, 3rd, and 5th stimuli, left and right bars were presented on the monitor for 500 ms, while auditory pure tones (1000 Hz, 500 ms, 70 dB) were presented simultaneously (see Fig. 1). For the 4th stimulus, in the apparent motion condition, left or right bars alternated for a duration of 33 ms with empty intervals (216 ms or 33 ms) between those two bars (i.e., visual apparent motion), while the two bars were presented for 500 ms in the static condition. Meanwhile, a single tone with varied duration (1000 Hz, 70 dB, randomly selected from 380, 450, 480, 520, 550, or 620 ms) was presented. By adjusting the interval between two bars, the pace of perceived visual motion in the fourth visual stimulus was altered: Slow apparent motion (interval of 216 ms), Fast apparent motion (interval of 33 ms), and Static condition. The speed of the Fast condition was 510 deg/s, and the Slow condition was 78 deg/s. The distance between two bars was kept constant.

#### Procedure

Participants sat in a dark room, and the distance between their eyes and the screen was kept at 57 cm. The trial sequence is shown in Fig. 1. In each trial, a fixation cross with a random duration between 700 and 900 ms was presented. At the end of each trial, participants judged whether the fourth sound was longer than the other four sounds. Before the fourth visual stimulus, the fixation cross changed to red to remind participants of the probe stimulus. There was no time limit for responses. Participants practised before the formal experiment. There were 192 trials for the Fast, Slow and Static conditions and 576 trials in total in the formal experiment.

#### Data analysis

**Figure 2:**
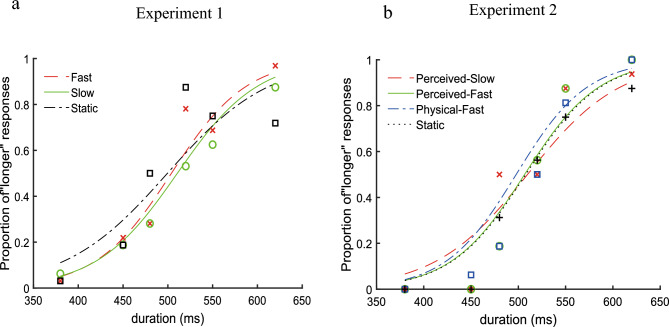
Psychometric function curves. (**a**) The averaged psychometric function curve in Experiment 1. The red dashed curve represents the Fast condition; the green solid curve represents the Slow condition; the black dot-dashed curve represents the Static condition. (**b**) The averaged psychometric function curve in Experiment 2. The red dashed curve represents the Perceived-Slow condition; the green solid curve represents the Perceived-Fast condition; the blue dot-dashed curve represents the Physical-Fast condition; the black dotted curve represents the Static condition.

The proportion that the comparison stimulus is perceived longer than the standard stimulus is plotted in Fig. 2a, in which the red dashed curve represents the Fast condition, the green solid curve represents the Slow condition, and the black dot-dashed curve represents the Static condition. Data were fitted to a logistic psychometric function^25^: p(long|X) = c + $\updelta$/(1 + exp((x-λ) × α)) using maximum likelihood methods. X is the probe duration, c is the lower limit of the correct response rate, $\updelta$ is the difference between the upper and lower limits of the correct response rate, λ is the bisection point, and α is the (negative) slope of the functions. We chose the best fitted function for each condition of each participant according to the maximum likelihood pseudo R^2^. Three participants were excluded from the analysis because their data in at least one condition could not be well fitted by the logistic psychometric functions (i.e., R^2^ < 0.1)^26^.

For each participant, the point of subjective equality (PSE) or the location parameter $\uplambda$ in the psychometric function was estimated as a by-product of model fitting. In essence, PSE represents the stimulus duration required to achieve the central proportion specified by the psychometric function (for example, 50% when the fitted lower and upper boundaries of correct response rates are 0% and 100%, respectively). Moreover, to indicate the direction of distortion for the perceived duration, shift rates of the PSE were also shown here using the following equation: Shift Rate (%) = (x − s)/s (x = PSE; s = standard duration, 500 ms)^27^. A negative shift rate value indicates that participants perceive the duration as longer than the standard duration (overestimation), while a positive shift rate means that participants perceive the duration as shorter than the standard duration (underestimation).

### Results

**Figure 3:**
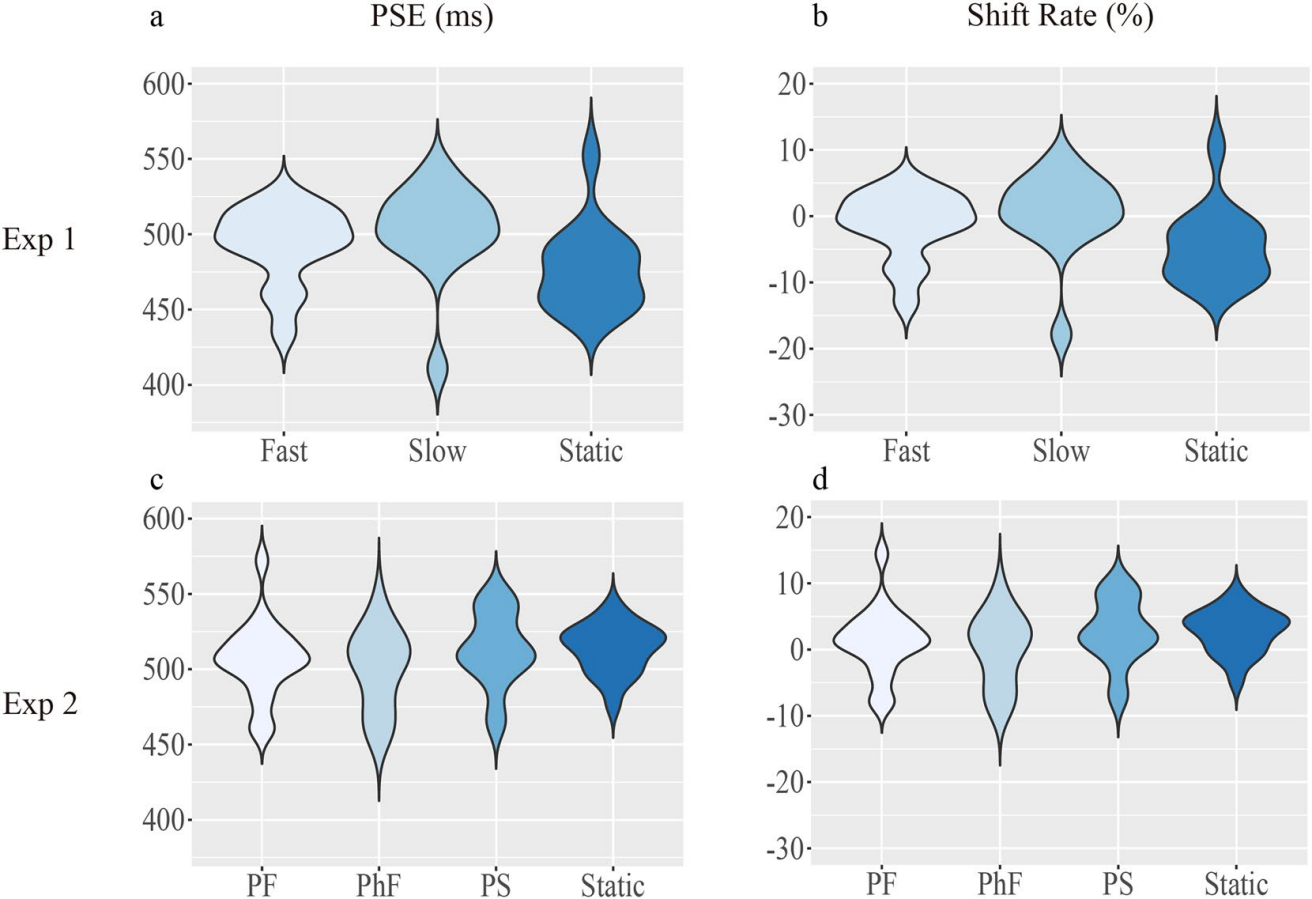
Distributions of PSE and shift rate in Experiments 1 and 2 (violin plots). (**a**) Skewed distributions of PSE (**a**) and shift rate (**b**) in Experiment 1. Symmetric distributions of PSE (**c**) and shift rate (**d**) in Experiment 2. PS represents the perceived-slow condition, PF represents the perceived-fast condition, and PhF represents the physical-fast condition.

**Figure 4:**
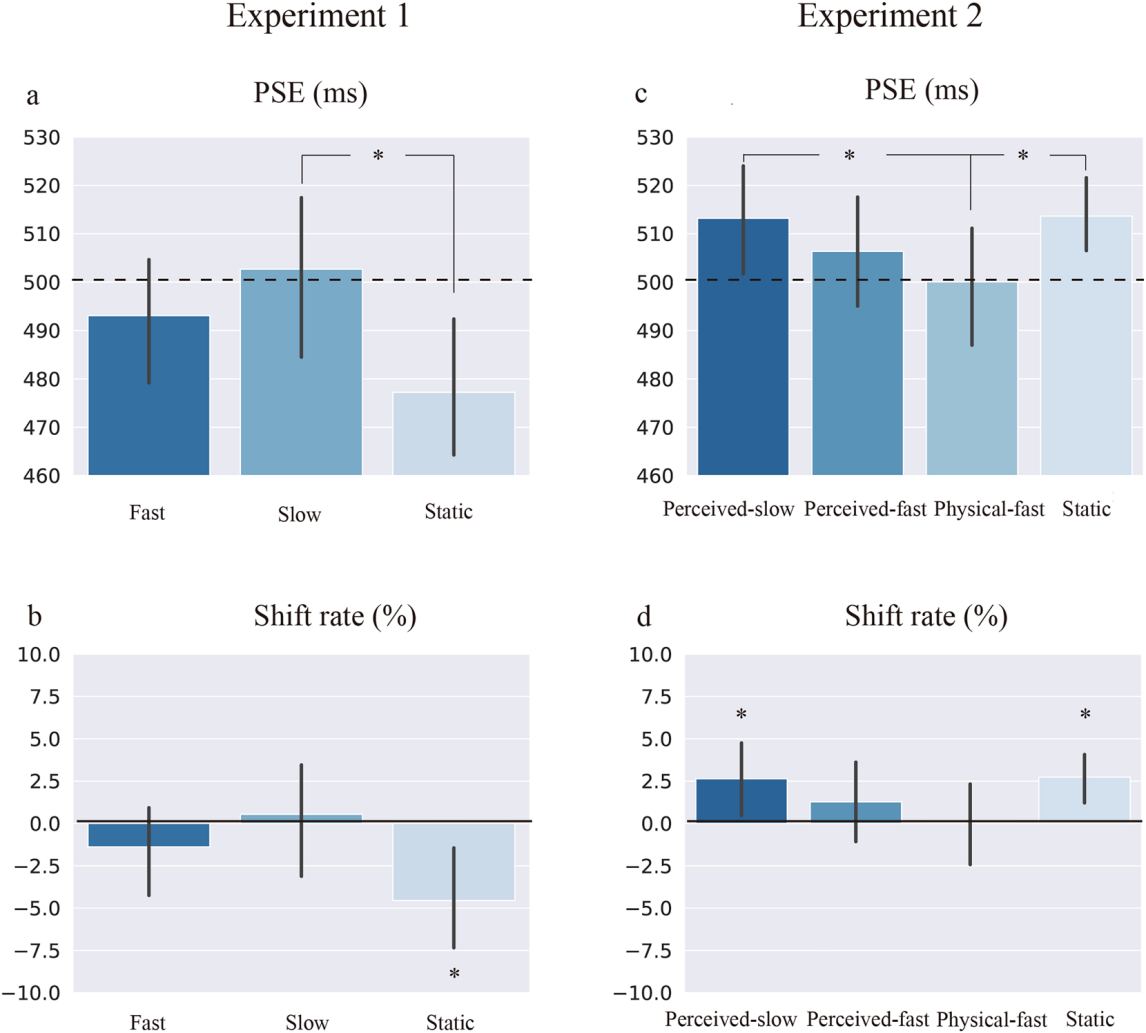
Mean PSE (**a**) and shift rate (**b**) of different experimental conditions in Experiment 1; mean PSE (**c**) and shift rate (**d**) of different experimental conditions in Experiment 2.

The distributions of PSE and shift rate are shown in Fig. 3. We implemented a one-way repeated ANOVA with the visual conditions as the independent variable for PSE in SPSS (22.0) (see Fig. 4a). For PSE, the main effect of motion was significant, *F*(2,26) = 6.63,* p* < 0.01, *η2* = 0.34, indicating that perception of auditory duration was modulated by simultaneous visual apparent motion. Further analysis (LSD corrected) showed that the mean PSE was significantly larger in the Slow condition (502.67 ms) than in the Static condition (477.22 ms, *t*(13) = 3.23, *p* < 0.01, *Cohen's d* = 0.85).

The shift rates of the average PSE for all three experimental conditions were calculated. Paired-samples *t* tests against zero were used for each condition, and LSD was used to adjust multiple comparisons^27^. The results showed that the PSE shift rate was significantly smaller than zero in the Static condition (*t*(13) = 3.01, *p* < 0.05, *Cohen's d* = 0.56), indicating that participants tended to overestimate the duration in only the Static condition (see Fig. 4b).

### Discussion

The results provide evidence that the duration perception of a tone could be distorted by the simultaneous visual apparent motion. The PSE results showed that participants tended to overestimate auditory duration under the Static condition compared with the Slow condition. These results are inconsistent with the SET model and previous studies^11,12^, which may be attributed to different stimulus combinations. In previous studies, only moving stimuli of different speeds were adopted, but no static condition was included. According to Ahrens and Sahani^28^, duration perception was modulated by two factors: internal estimation and sensory-based estimation. Internal estimation refers to the estimation of the time interval without sensory input, while sensory-based estimation refers to internally integrated processes derived from the sensory input, by which one can estimate the time interval. In the present study, the judgement of duration in the Static condition mainly relied on internal estimation. In contrast, duration judgement in apparent motion conditions (whether it is Fast or Slow) can use both internal estimation and sensory-based estimation since (additional) continuous sensory input could be used. Thus, the time perception should be more accurate in the apparent motion conditions, i.e., the time dilation effect would be weakened, since both systems of time estimation are functioning. However, it remains unclear whether such time distortion is caused by perceived speed or temporal frequency^9,12^, which is examined in Experiment 2.

## Experiment 2

In Experiment 1, we observed that visual apparent motion distorted the perception of auditory duration. In order to separate the two interrelated variables of speed and temporal frequency, manipulations were made to both the perceived speed and temporal frequency of visual apparent motion. This enabled us to determine whether any distortion was caused by motion speed or temporal frequency.

### Method

#### Participants

Twenty college students (10 males, mean age 20.4 years, range 18–23 years, SD 1.86 years) took part in the experiment. All participants who reported normal hearing and normal or corrected-to-normal vision participated in the experiment. They signed informed consent before the experiment and were paid for participation. The study was conducted in accordance with the guidelines in the Declaration of Helsinki (2000) and approved by the Ethics Committee of the Department of Psychology, Sun Yat-sen University.

#### Stimuli and apparatus

**Figure 5:**
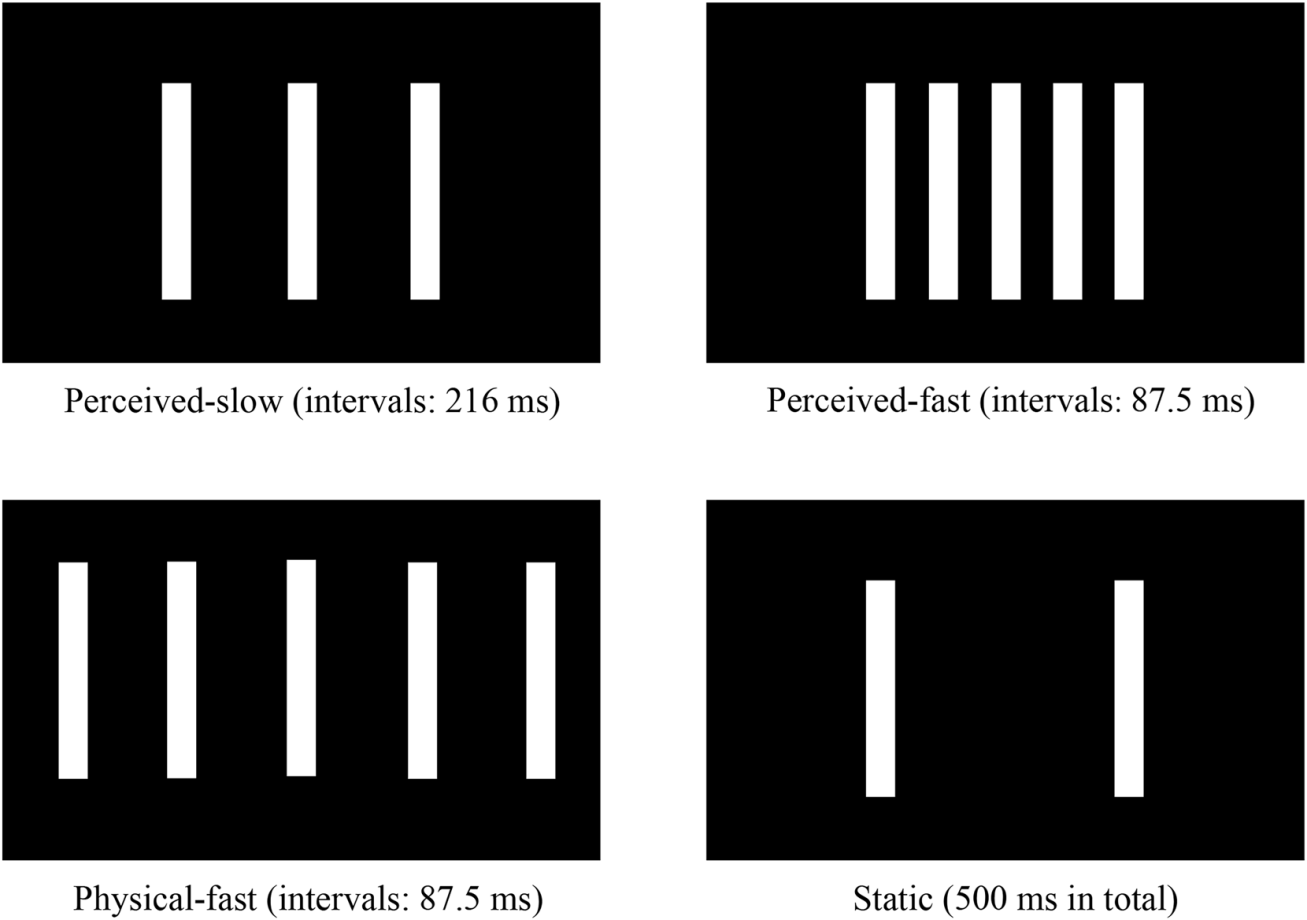
The apparent motion stimuli used in Experiment 2. In the Perceived-slow condition, three bars appear in alternation for 33 ms with empty intervals of 216 ms. Five bars appear in alternation for 33 ms with empty intervals of 87.5 ms in the Perceived-fast condition, while with intervals of 87.5 ms in the Physical-fast condition. Static condition is the same as that in Experiment 1.

The visual and auditory stimuli in Experiment 2 were the same as those in Experiment 1, except for the fourth visual stimulus. There were four kinds of visual stimuli (see Fig. 5): Perceived-slow apparent motion consisted of three bars appeared in alternation for 33 ms with empty intervals of 216 ms; Perceived-fast apparent motion consisted of five bars appeared in alternation for 33 ms with empty intervals of 87.5 ms; Physical-fast apparent motion also consisted of five bars appeared in alternation for 33 ms with empty intervals of 87.5 ms; Static condition consisted of two vertical bars that were separated by a distance (14° visual angle) and lasted 500 ms. The speed of the Physical-fast condition (32 deg/s) was twice as fast as that of the Perceived-slow condition and Perceived-fast condition (16 deg/s), i.e., the motion path in the Physical-fast condition was twice as long as that in the Perceived-slow and Perceived-fast conditions. Moreover, the temporal frequencies of the Physical-fast condition and Perceived-fast condition were the same, which were faster than that of the Perceived-slow condition. Each bar was presented sequentially from left to right in all the apparent motion conditions. To avoid the possible confounding of moving direction, the directions of apparent motion were maintained from left to right across all experimental conditions.

#### Procedure

The trial sequence and experimental settings were the same as in Experiment 1. Each visual condition (Perceived-slow, Perceived-fast, Physical-fast, and Static) was repeated 96 times. The visual condition was presented in blocks, and the order was counterbalanced across participants. Each participant completed 384 trials in total in the formal experiment.

#### Data analysis

The proportion that the probe stimulus is perceived as longer than the standard stimulus is plotted in Fig. 2b, in which the red dashed curve represents the Perceived-slow condition, the green solid curve the Perceived-fast condition, the blue dot-dashed curve the Physical-fast condition, and the black dotted curve the Static condition. Data were averaged across participants for each experimental condition and fitted to logistic psychometric functions: p(long|X) = 1/(1 + e((x − λ) × α)). X is the probe duration, λ is the bisection point, and α is the (negative) slope of the functions. Two participants were excluded from the final analysis due to R^2^ < 0.1^20^. The point of subjective equality (PSE) was calculated for each experimental condition.

### Results

We implemented an one-way repeated ANOVA with visual apparent motion conditions as the independent variable for PSE in SPSS (22.0). The main effect of visual apparent motion was significant, *F*(3,51) = 3.04, *p* < 0.05, *η2* = 0.15, indicating that perception of auditory duration was affected by the simultaneous visual apparent motion. Post hoc paired *t* tests (LSD corrected) showed that the mean PSE was significantly smaller in the Physical-fast condition (500.06 ms) than in the Static (513.67 ms, *t*(17) = -3.039, *p* < 0.01, *Cohen's d* = 0.61) and the Perceived-slow (513.20 ms, *t*(17) = 2.433, *p* < 0.05, *Cohen's d* = 0.51) conditions (see Fig. 4c), indicating that participants underestimated the perceived duration of the auditory target in the Static and the Perceived-slow conditions than in the Physical-fast condition.

The shift rates of the average PSE were calculated^27^. Paired-samples *t* tests against zero showed that the shift rates of PSE were significantly larger than zero in the Static (*t*(17) = 3.54, *p* < 0.01, *Cohen's d* = 0.65) and the Perceived-slow conditions (*t*(17) = 2.26, *p* < 0.05, *Cohen's d* = 0.48) (see Fig. 4d). The results suggested that visual apparent motion could induce perceptual distortion of auditory duration, as observed in the Static and the Perceived-slow condition. Participants tended to underestimate auditory duration in Static and Perceived-slow conditions but not in Perceived-fast and Physical-fast conditions.

### Discussion

Experiment 2 further examined whether the auditory time distortion influenced by visual apparent motion was caused by perceived speed or temporal frequency. For the PSE results, there was a significant difference between the Perceived-slow and Physical-fast conditions, in which both the temporal frequency (flashing rate of bars) and speed are different. Thus, our results imply that both the temporal frequency and speed of visual apparent motion are vital for the interval perception of auditory tones. These results are partly in line with previous findings, in which they demonstrated that the time distortion should be attributed to the temporal frequency^12^ or the speed^9^. In addition, the temporal frequencies differed between the Perceived-slow and Perceived-fast conditions, while the speeds of apparent motion were the same for these two conditions (the bars moved the same distance during the same interval). No significant difference was observed between the Perceived-slow and Perceived-fast conditions. Similarly, for the Perceived-fast and Physical-fast conditions, temporal frequencies were the same, while the speeds of apparent motion were different (as the bar moved the same distance at a certain period of duration). Again, no significant difference was observed between those two conditions. Therefore, these results indicate that the temporal frequency and speed jointly modulates the perception of auditory duration together.

## General discussion

In the present study, we investigated whether visual apparent motion could modulate time perception in the auditory modality. Moreover, we explored whether perceived auditory duration was affected by the speed or temporal frequency of visual apparent motion. In Experiment 1, the mean PSE was significantly larger in the Slow condition than in the Static condition. Moreover, participants overestimated the duration in only the Static condition, which was not observed under the apparent motion conditions. In Experiment 2, the PSE in the Physical-fast condition was significantly smaller than those in the Perceived-slow and the Static conditions. Moreover, the participants underestimated the time duration in the Perceived-slow and Static conditions but not in the Perceived-fast and Physical-fast conditions.

Most importantly, a significant difference in mean PSE was found between the Physical-fast condition and the Perceived-slow condition in Experiment 2, indicating that auditory time perception is not modulated solely by the temporal frequency or by the speed of visual apparent motion, and the two factors comodulate the duration perception in the auditory modality. Kaneko and Murakami found that speed is the key factor in modulating time perception^9^. Conversely, Kanai et al. found that the time dilation effect mainly depended on the temporal frequency of the motion stimuli^12^. Such discrepancies might be due to the saliency of the stimuli in these two studies. More precisely, in Kanai et al., the visual stimuli were the expanding gratings in which the temporal frequency information was salient^12^^.^ Thus, one could judge time duration based on temporal frequency. In contrast, compared with apparent motion stimuli, the Gabor patch used by Kaneko and Murakami was more salient in speed^9^. However, in the present study, visual apparent motion was used, in which the information of both temporal frequency and speed are salient compared with previous studies^9,12^. Therefore, speed and temporal frequency both modulated the perception of auditory duration in the present study.

**Figure 6:**
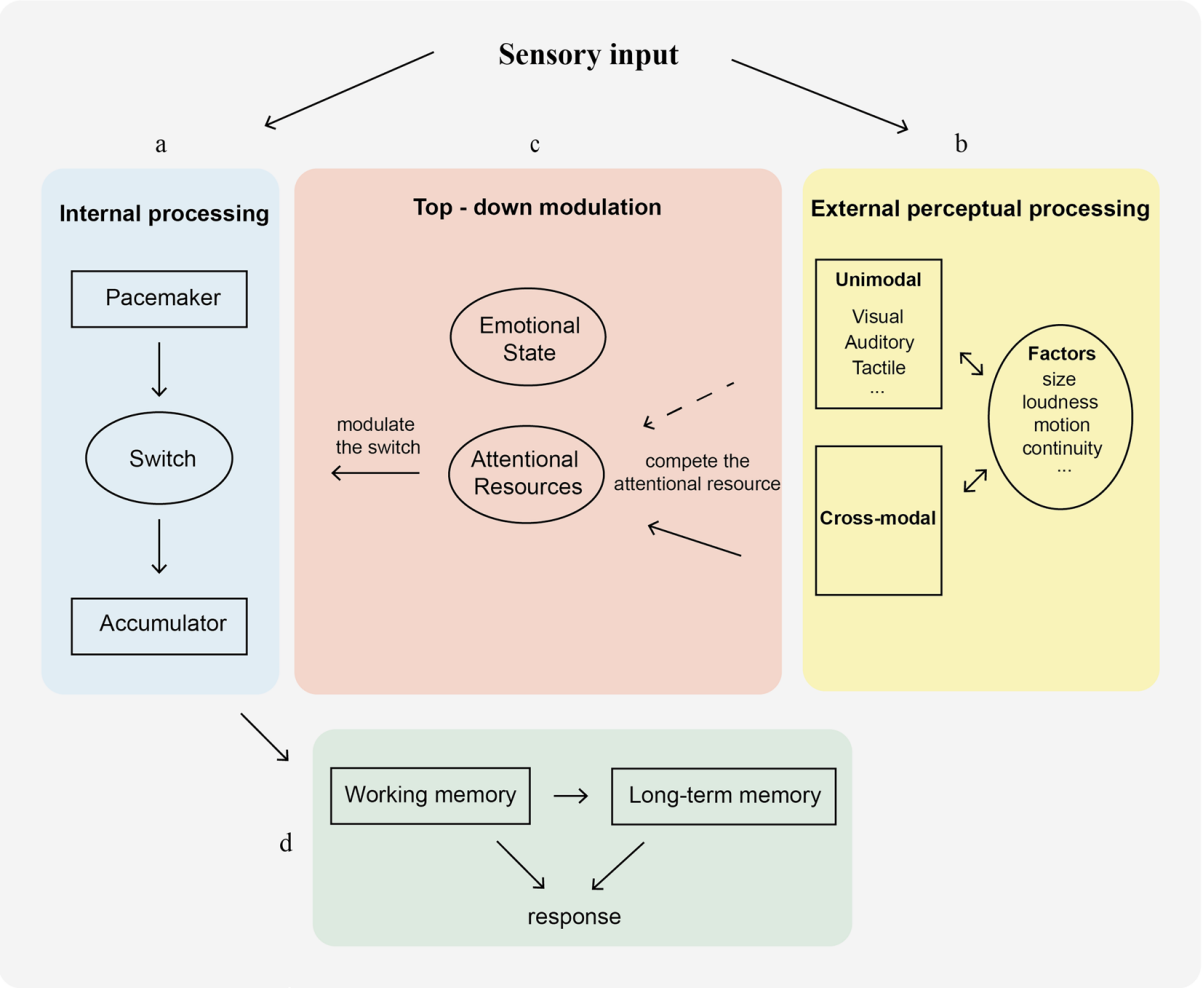
A modified SET model. (**a**) The internal processing module, also known as the original SET model (depicted with light blue background). It consists a dedicated pacemaker which emits pulses, the switch as a gate that modulates how many pulses flow into an accumulator. (**b**) The external perceptual processing module (depicted with light yellow background) consists two types of perceptual representations, unimodal and cross-modal representation. Both of them are affected by some factors, such as size, loudness, motion, continuity, et al. (depicted within the circle of this module). (**c**) The top-down modulation module (depicted with light red background) connects the internal processing module and the external perceptual processing module. The attentional resource submodule (depicted within the circle of this module) plays a key role, which is mainly responsible for modulating the allocation of attention resources. For example, external perceptual representations compete the limited attentional resource with the switch of the internal processing. Specifically, cross-modal stimuli would compete for more attention resources than unimodal stimuli (the compete processing of cross-modal stimuli indicated by solid line, and unimodal by dash line). (**d**) The response processing module. It is responsible for transferring the accumulated pulses to form the representation of subjective duration in the accumulator to working memory and some of the representation of subjective duration to the long-term memory; the judgement of a duration was based on comparison of the working memory representation with previously encoded representation of subjective duration.

According to the scalar expectancy theory (SET)^1,2^, there exists a dedicated pacemaker that generates and emits pulses into the accumulator. Before the pulses enter the accumulator, a switch modulates the number of pulses flowing into the accumulator. The accumulated pulses form the representation of subjective duration in the accumulator, which transfers to working memory. Then, the judgement of a duration was based on comparison of the representation in the working memory with a previous representation in the long-term memory. External perceptual stimuli altered the rate of the pacemaker and then affected the subjective duration (shown in Fig. 6a,d). In the present study, features from both visual and auditory modalities need to be processed. According to previous studies, attention could be attracted by multisensory stimuli more easily than unisensory stimuli^29^, even when multisensory stimuli act as distractors^30^. Thus, returning to this modified SET model, the switch module controls the number of pulses emitted from the pacemaker to the accumulator, which is modulated by attention. The present study found that the processing of external stimuli from multiple senses competes for attentional resources and affects the switching mechanism, resulting in fewer pulses being emitted into the accumulator. This reduces the time distortion effect predicted by the SET model. In contrast, unimodal stimuli compete less for attentional resources than multisensory stimuli, resulting in a stronger time distortion effect (as shown in Fig. 6b,c).

Our findings indicate that the time compression effect occurred under apparent motion conditions in Experiment 2 but not in Experiment 1. These findings could be attributed to different motion types being perceived between Experiments 1 and 2. The visual bars moved following a circular motion trajectory in Experiment 1, which is likely to be perceived as an independent object and regarded as global motion, similar to the stimuli in the study of Yamamoto and Miura^31^. In contrast, the motion trajectory of the stimuli in Experiment 2 is linear, e.g., moving from left to right in the horizontal direction, which is more likely to be perceived as local motion, similar to the study of Kanai et al.^12^. Further research could be done to investigate how motion type could modulate time perception.

Furthermore, two vertical bars, static or in motion, were utilized as visual stimuli in the current research. If a blank screen was utilized as the control condition, the presentation of visual stimuli that appear to move might be perceived by the participants in an abrupt and unpredictable manner, which is quite noticeable when compared to two visually static bars. As a result, we used two static visual bars as the control condition in this study. In future studies, a control condition without any visual stimulation could also be incorporated. Additionally, previous studies have demonstrated that the auditory modality dominates the visual modality when it comes to processing temporal information^32^. Future research could delve deeper into how auditory stimuli impact the perception of visual duration.

One may argue that the time dilation effect in the Static condition was found in Experiment 1, while the time compression effect was found in the static condition in Experiment 2. These seemingly paradoxical results may be due to the mechanism of attention resource allocation. According to the processing principle of subjective time^33^, the perception of an object can be roughly divided into two parts of processing: one is the processing of the object itself, and the other is processing the temporal information of that object. If more attention resources are allocated to temporal processing, the time judgement performance improves. In the present study, compared with apparent motion conditions, participants may place less attention resources into the static stimuli, thus making the temporal productions in the static condition more variable. This idea could be supported by a recent study in which the researchers found that duration judgements were systematically shorter and more variable with higher cognitive load^34^.

According to Freeman and Driver's research^24^, one would anticipate observing a time dilation or compression aftereffect of apparent motion. Their experiment included two phases for each trial: a 30-s exposure phase and a 5-s after-effect phase. In contrast, the current study did not incorporate an after-effect measurement phase. As a result, any potential after-effect generated by preceding visual apparent motion stimuli may have been overwritten by the current visual bars. Additionally, in the present study, the duration judgment task was conducted immediately after the display of visual apparent motion stimuli, with no delay, which means that the after-effect of apparent motion would not influence the perception of duration in the auditory modality.

To conclude, for the subsecond duration, auditory time perception can be modulated by simultaneous visual apparent motion. Furthermore, the perception of auditory duration can be jointly influenced by both the speed and temporal frequency of visual apparent motion. Subsequent research can delve deeper into the co-modulation of auditory duration perception by varying the speed and temporal frequency of apparent motion more comprehensively.

## Supplementary Information

Supplementary Information.

## Data Availability

Datasets used in the present study are available in the online repositories at https://osf.io/mzwg8/.

## References

[1] Gibbon J, Church RM, Meck WH (1984). Scalar timing in memory. Ann. N Y Acad. Sci..

[2] Treisman M (1963). Temporal discrimination and the indifference interval: Implications for a model of the "internal clock". Psychol. Monogr..

[3] Ono F, Kawahara J (2007). The subjective size of visual stimuli affects the perceived duration of their presentation. Percept. Psychophys..

[4] Xuan B, Zhang D, He S, Chen X (2007). Larger stimuli are judged to last longer. J. Vis..

[5] Aaen-Stockdale C, Hotchkiss J, Heron J, Whitaker D (2011). Perceived time is spatial frequency dependent. Vis. Res..

[6] Lee KH, Seelam K, O'Brien T (2011). The relativity of time perception produced by facial emotion stimuli. Cogn. Emot..

[7] Herbst, S.K., Javadi, A.H., van der Meer, E., & Busch, N.A. How long depends on how fast--perceived flicker dilates subjective duration. *PLoS One*. **8**(10), e76074 (2013). 10.1371/journal.pone.0076074 PMC3806760 24194829

[8] Linares D, Gorea A (2015). Temporal frequency of events rather than speed dilates perceived duration of moving objects. Sci. Rep..

[9] Kaneko S, Murakami I (2009). Perceived duration of visual motion increases with speed. J Vis..

[10] Gorea A, Kim J (2015). Time dilates more with apparent than with physical speed. J Vis..

[11] Yamamoto K, Miura K (2012). Perceived duration of plaid motion increases with pattern speed rather than component speed. J Vis..

[12] Kanai R, Paffen CL, Hogendoorn H, Verstraten FA (2006). Time dilation in dynamic visual display. J Vis..

[13] Fendrich R, Corballis PM (2001). The temporal cross-capture of audition and vision. Percept. Psychophys..

[14] Morein-Zamir S, Soto-Faraco S, Kingstone A (2003). Auditory capture of vision: Examining temporal ventriloquism. Cogn. Brain Res..

[15] Vroomen J, de Gelder B (2004). Temporal ventriloquism: Sound modulates the flash-lag effect. J. Exp. Psychol. Hum. Percept. Perform..

[16] Barne LC (2018). A common representation of time across visual and auditory modalities. Neuropsychologia.

[17] Chen KM, Yeh SL (2009). Asymmetric cross-modal effects in time perception. Acta Psychol..

[18] Yue Z, Gao T, Chen L, Wu J (2016). Odors bias time perception in visual and auditory modalities. Front. Psychol..

[19] Chen Y, Huang X, Luo Y, Peng C, Liu C (2010). Differences in the neural basis of automatic auditory and visual time perception: ERP evidence from an across-modal delayed response oddball task. Brain Res..

[20] Bratzke D, Ulrich R (2019). Temporal reproduction within and across senses: Testing the supramodal property of the pacemaker-counter model. J. Exp. Psychol. Hum. Percept. Perform..

[21] Goebel R, Khorram-Sefat D, Muckli L, Hacker H, Singer W (1998). The constructive nature of vision: Direct evidence from functional magnetic resonance imaging studies of apparent motion and motion imagery. Eur. J. Neurosci..

[22] Vetter P, Grosbras MH, Muckli L (2015). TMS over V5 disrupts motion prediction. Cereb. Cortex..

[23] Ramachandran VS, Anstis SM (1986). The perception of apparent motion. Sci. Am..

[24] Freeman E, Driver J (2008). Direction of visual apparent motion driven solely by timing of a static sound. Curr. Biol..

[25] Wichmann FA, Hill NJ (2001). The psychometric function: I. Fitting, sampling, and goodness of fit. Percept. Psychophys..

[26] Ellis PD (2010). The essential guide to effect sizes: Statistical power, meta-analysis, and the interpretation of research results.

[27] Yuasa K, Yotsumoto Y (2015). Opposite distortions in interval timing perception for visual and auditory stimuli with temporal modulations. PLoS ONE.

[28] Ahrens MB, Sahani M (2011). Observers exploit stochastic models of sensory change to help judge the passage of time. Curr. Biol..

[29] Santangelo V, Spence C (2007). Multisensory cues capture spatial attention regardless of perceptual load. J. Exp. Psychol. Hum. Percept. Perform..

[30] Yuan Y, He X, Yue Z (2023). Working memory load modulates the processing of audiovisual distractors: A behavioral and event-related potentials study. Front. Integr. Neurosci..

[31] Yamamoto K, Miura K (2016). Effect of motion coherence on time perception relates to perceived speed. Vis. Res..

[32] Lukas, S., Philipp, A. M. & Koch, I. Crossmodal attention switching: Auditory dominance in temporal discrimination tasks. *Acta Psychol. ***153**, 139–146 (2014). 10.1016/j.actpsy.2014.10.003 25463554

[33] Matthews WJ, Meck WH (2016). Temporal cognition: Connecting subjective time to perception, attention, and memory. Psychol. Bull..

[34] Block RA, Hancock PA, Zakay D (2010). How cognitive load affects duration judgments: A meta-analytic review. Acta Psychol..

