# Supplementary material for: The speed and temporal frequency of visual apparent motion modulate auditory duration perception
Source: Sci Rep. 2023 Jul 12;13:11281. doi: 10.1038/s41598-023-38183-w (PMC10338538; doi:10.1038/s41598-023-38183-w)
Supplement: Supplementary file 1 — Supplementary Information. [file 41598_2023_38183_MOESM1_ESM.pdf]

The speed and temporal frequency of visual apparent motion modulate auditory duration perception

Xiang He[1], Zijun Ke[1], Zehua Wu[1], Lihan Chen[2*], Zhenzhu Yue[1*]

[1]Department of Psychology, Sun Yat-sen University, Guangzhou, 510006, China

[2]School of Psychological and Cognitive Sciences and Beijing Key Laboratory of Behavior and Mental Health, Peking University, Beijing, 100871, China

Running title: visual apparent motion affects auditory timing

Address correspondence to:
Dr. Zhenzhu Yue, Department of Psychology, Sun Yat-sen University
Email: yuezhzh@mail.sysu.edu.cn

Or to Dr. Lihan Chen, Email: clh@pku.edu.cn

1. **Data distribution in Experiments 1 and 2 (see Supplementary Figure 1)**

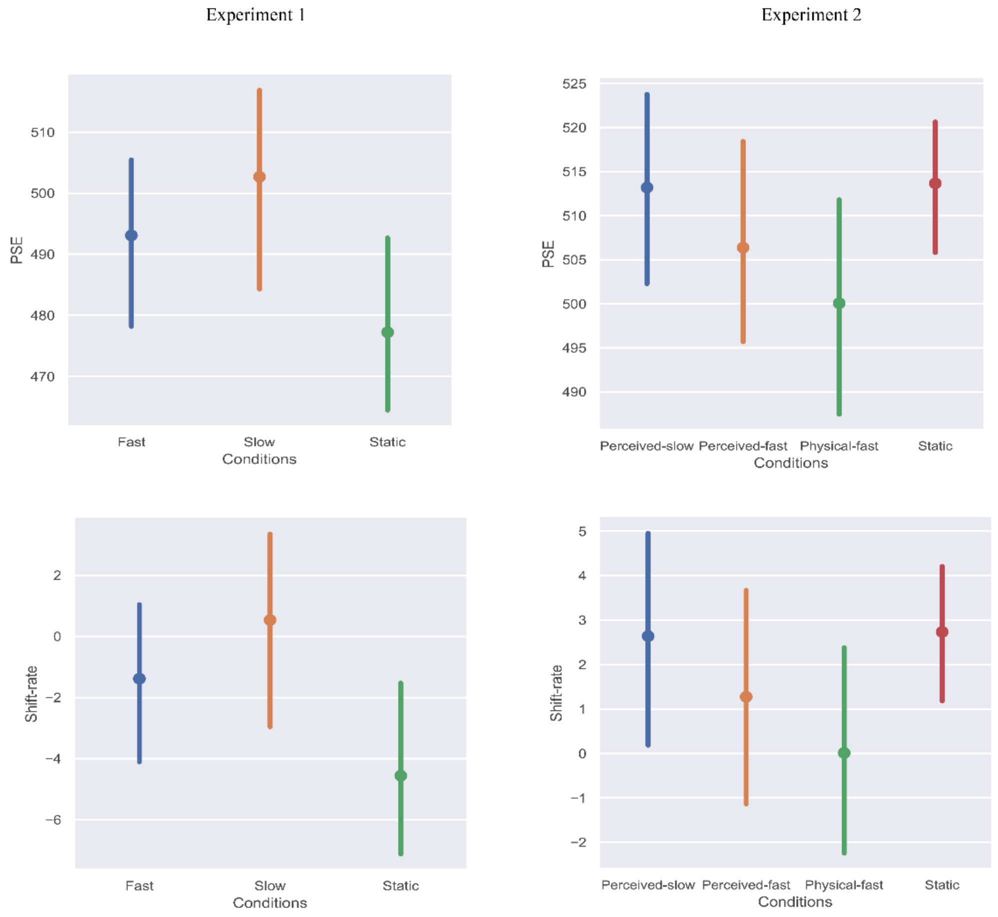

Supplementary Figure 1 Means and 95% CIs for Experiments 1 and 2

2. Results of Shapiro-Wilk test in Experiments 1 and 2

Shapiro-Wilk tests in Experiments 1 and 2 show that significance levels of Fast and Slow conditions in Experiment 1 are less than 0.1, indicating that the data in these two conditions are in a skewed distributions. By contrast, the data in Experiment 2 are in symmetric distributions (detailed information see Supplementary Table 1 and 2)

Supplementary Table 1 The results of Shapiro-Wilk test in Experiment 1

	Value	Df	Significance
Fast	.905	14	.134
Slow	.840	14	.016
Static	.877	14	.053

Supplementary Table 2 The results of Shapiro-Wilk test in Experiment 2

	Value	Df	Significance
Perceived-slow	.928	18	.182
Perceived-fast	.936	18	.244
Physical-fast	.948	18	.388
Static	.964	18	.677

3. Non-parametric tests in Experiments 1 and 2

Non-parametric tests were applied in Experiments 1 and 2, including Friedman test and Wilcoxon test. Detailed results could be found as following (see Supplementary Tables 3-8), and these results of non-parametric test echo the original AVOVA results.

Supplementary Table 3 The results of Friedman test for PSE in Experiment 1

	N	X^2	Df	Significance
PSE	14	9.143	2	0.010
Shift rate	14	9.143	2	0.010

Supplementary Table 4 The results of Friedman test for PSE in Experiment 2

	N	X^2	Df	Significance
PSE	18	4.20	3	0.241
Shift rate	18	4.20	3	0.241

Supplementary Table 5 The results of Wilcoxon test for PSE in Experiment 1

	Slow - Fast	Static - Fast	Static - Slow
Z	-1.664	-2.040	-2.542
Significance	.096	.041	.011 *

Supplementary Table 6 The results of Wilcoxon test for Shift rate in Experiment 1

	Fast-Zero	Slow-Zero	Static-Zero
Z	-.568	-2.556	-1.079
Significance	.570	.011 *	.281

Supplementary Table 7 The results of Wilcoxon test for Shift Rate in Experiment 2

	Z	Significance
Perceived-fast - Perceived-slow	-.980	.327
Physical-fast – Perceived-slow	-2.286	.022 *
Static – Perceived-slow	-.022	.983
Physical-fast – Perceived-fast	-.936	.349
Static – Perceived-fast	-1.241	.215
Static – Physical-fast	-2.765	.006 **

Supplementary Table 8 The results of Wilcoxon test for Shift Rate in Experiment 2

	Z	Significance
Perceived-fast – Zero	-1.328	.184
Physical-fast – Zero	-.024	.981
Perceived-slow – Zero	-2.107	.035 *
Static – Zero	-2.678	.007 **

4. Estimation tests in Experiments 1 and 2

We did the estimation statistics using the Exploratory Software for Confidence Intervals (ESCI)[1]. The results of Experiment 1 are showed in Supplementary Table 9, and we find a significant difference for PSE between Slow and Static conditions. No significant differences are found between other conditions. The results of estimation test in Experiment 1 are consistent with the repeated measure ANOVA (RM ANOVA) in the revised manuscript.

Similarly, the results of estimation test in Experiment 2 are also consistent with the RM ANOVA in the manuscript. The results of Experiment 2 are showed in Supplementary Table 10, and we find a significant difference between Perceived-slow and Physical-fast conditions. Besides, we also observe a significant difference between Physical-fast and Static conditions. No significant differences are found between other conditions.

Since shift rate is the linear transformation of PSE, the results of estimation test for shift rate and PSE should be exactly the same. To avoid redundancy, only the results of PSE are reported here.

[1] Cumming, Geoff, and Robert Calin-Jageman. *Introduction to the new statistics: Estimation, open science, and beyond*. Routledge, 2016.

Supplementary Table 9 The results of estimation test for PSE in Experiment 1

		95 % CI (difference)			
Condition	M (difference)	Lower	Upper	s (difference)	*p*
Fast - Slow	9.60	-1.22	20.4	18.7	0.0775
Fast – Static	15.9	-1.24	33.0	29.6	0.0664
Slow -Static	25.5	8.46	42.5	29.5	0.0065 **

Supplementary Table 10 The results of estimation test for PSE in Experiment 2

		95 % CI (difference)			
Condition	M (difference)	Lower	Upper	s (difference)	*p*
PS - PF	6.82	-6.20	19.8	26.2	0.284
PS - PhF	13.1	1.74	24.5	22.9	0.026 **
PS – Static	-0.471	-11.6	10.7	22.4	0.930
PF – PhF	6.32	-3.67	16.3	20.1	0.200
PF – Static	-7.29	-18.1	3.51	21.7	0.173
PhF – Static	-13.6	-23.1	-4.16	19.0	0.007 **

Note：PS represents the perceived-slow condition, PF represents the perceived-fast condition, and PhF represents the physical-fast condition.
